# Patellar Osteoid Osteoma as a Cause of Anterior Knee Pain in Adolescents: A Case Report and Literature Review

**DOI:** 10.1155/2013/746472

**Published:** 2013-03-10

**Authors:** Claudio Chillemi, Vincenzo Franceschini, Massimiliano D'Erme, Giorgio Ippolito, Pasquale Farsetti

**Affiliations:** ^1^Department of Orthopaedics and Traumatology, Istituto Chirurgico Ortopedico Traumatologico (ICOT), Via Faggiana 1668, 04100 Latina, Italy; ^2^Department of Orthopaedics and Traumatology, Sapienza University of Rome, ICOT, Via Faggiana 1668, 04100 Latina, Italy; ^3^Department of Radiology, Istituto Chirurgico Ortopedico Traumatologico (ICOT), Via Faggiana 1668, 04100 Latina, Italy; ^4^Department of Orthopaedics, University of Rome Tor Vergata, Via del Politecnico 1, 00133 Rome, Italy

## Abstract

Anterior Knee Pain (AKP) is an important cause of complaint in adolescents which can suggest many possible diseases. Scientific literature concerning this complex symptom is wide and diversified. We report a rare case of patellar osteoid osteoma which affected a thirteen-year-old female who had suffered from anterior left knee pain for almost six months. The diagnosis was suspected from an accurate anamnesis, a careful clinical examination, and confirmed by imaging. Several minimally invasive techniques can be employed to treat osteoid osteoma. However, we consider CT-guided percutaneous drilling the safest and most effective procedure in case of patellar location. Despite its rarity, patellar osteoid osteoma ranges in the differential diagnosis for all patients suffering from AKP.

## 1. Introduction 

Anterior knee pain (AKP) is one of the most common musculoskeletal disorders and an important source of disability with a prevalence ranging 15–33% in the active adult population and 21–45% in adolescents [1]. Each year 54% of the young athletes seen in primary care setting complain of some degree of knee pain [1].

 AKP is characterised by a set of symptoms defined in the past with the generic term of “chondromalacia patellae” [1]. This concept was later abandoned because it had no diagnostic, therapeutic, or prognostic implication [2]. The underlying etiology of AKP has been extensively studied, and many possible causes have been identified [3]. The patient should always be questioned about previous knee trauma and surgery [1]. An accurate characterization of the knee pain (in terms of location, radiation, qualities, and exacerbating or relieving factors/activities) is very helpful in focusing on the diagnosis as well as a careful clinical examination and an appropriate imaging [1].

The most common causes of AKP in children and adolescents with no history of trauma are patellofemoral malalignment, tibial apophysitis (i.e., Osgood-Schlatter disease), patellar tendonitis, and less frequently slipped capital femoral epiphysis and osteochondritis dissecans [4]. 

Literature data reports several other causes of AKP described as less frequent but which should be taken into account for a correct differential diagnosis such as septic arthritis, synovial impingement, and tumors [1, 2].

The aim of this paper is to report a rare case of patellar osteoid osteoma as a cause of AKP and its treatment along with a review of the literature.

## 2. Case Report

**Figure 1:**
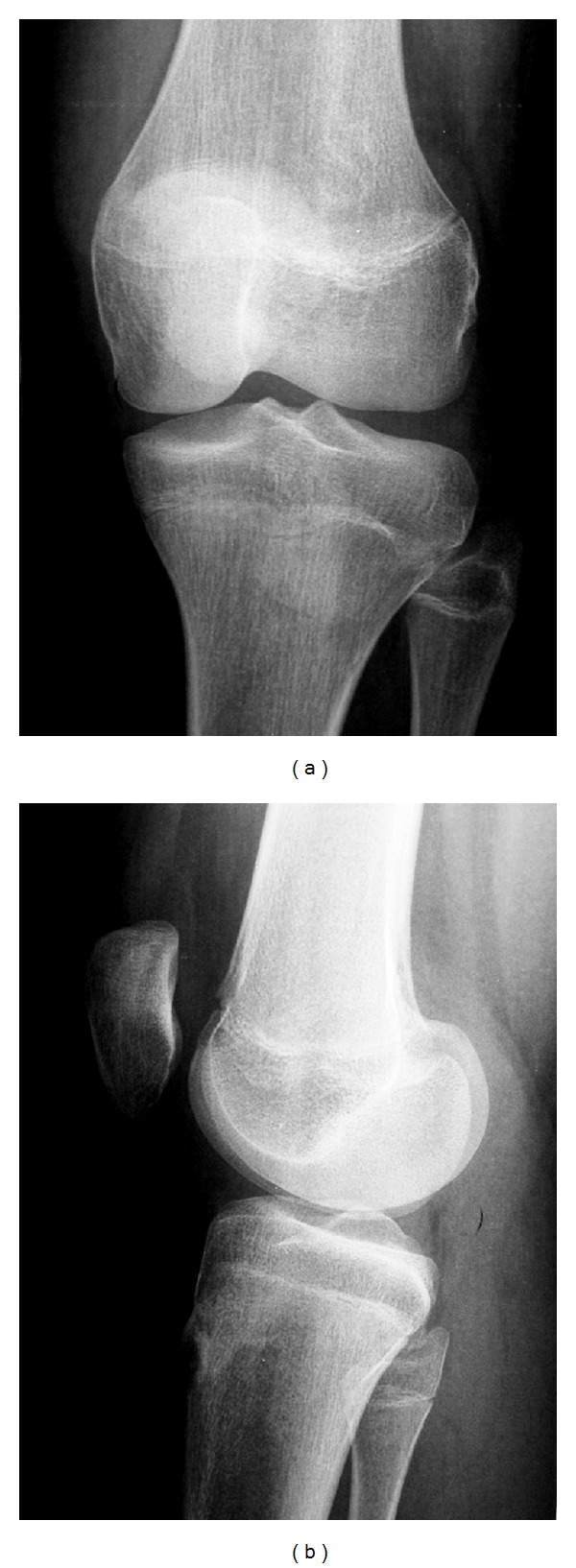
Radiograph of the patient's left knee in anteroposterior (a) and lateral (b) view.

**Figure 2:**
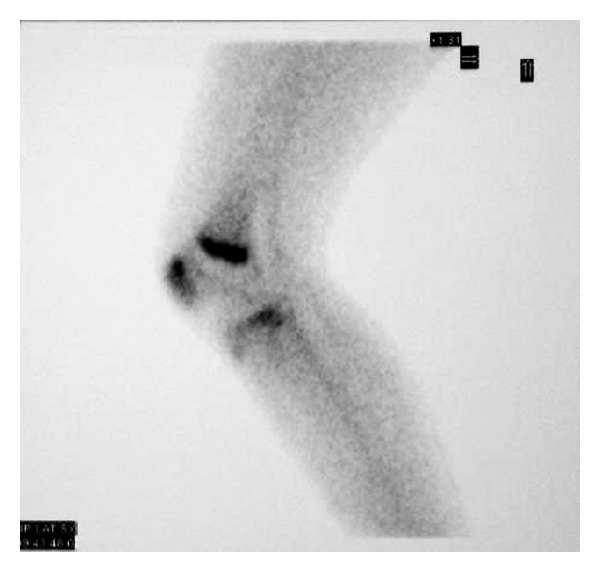
Three-phase scintigraphy showing a picking up area on the superior part of the patella.

**Figure 3:**
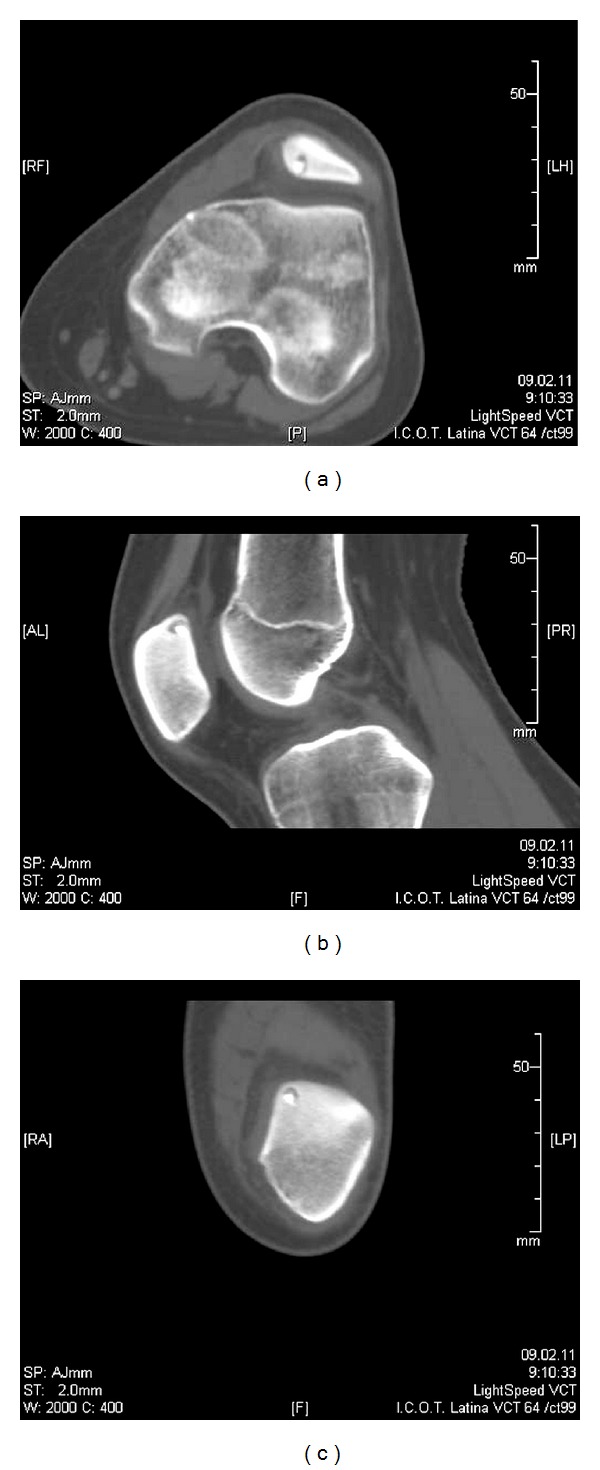
(a) Axial, (b) sagittal, and (c) coronal CT scans show a round, well-marginated sclerotic lesion of about 8 mm with a hypodense rim and a centrally calcified nidus.

A thirteen-year-old female was referred to our department with a six-month history of intense anterior left knee pain. The pain was localized at the anterosuperior part of the knee. It was described as continuous and referred to be 3 on the visual analogue scale (VAS) [5] with exacerbation during sport activity until a value of 7. During the last month, the pain worsened especially at night reaching a value of 8 on the VAS without any exacerbation during daily activity. The patient had sought medical attention for the first time six months earlier when she was diagnosed with a PFM and underwent a course of physical therapy without any benefit. Her past medical history was unremarkable and no previous trauma was reported. There was no fever or any other systemic findings. Physical examination revealed a normal Q angle with a complete and painless range of motion (ROM). Tenderness during palpation of the superior part of the patella at the quadriceps insertion point was noted. There was no patellar crepitus and the patellar apprehension test was negative. Laboratory studies including erythrocyte sedimentation rate, C-reactive protein, and basic biochemical tests did not show any abnormality. A previous plain radiograph of the patient's knee in two projections was considered to be negative (Figure 1). Considering that the pain was especially nocturnal, the patient was asked to undertake a course of conservative treatment, which consisted of taking acetyl-salicylic acid (one aspirin) every night for a period of ten days. At followup, the patient reported significant pain reduction every time she took aspirin. A three-phase scintigraphy was taken and it revealed a picking up area on the superior part of the left patella (Figure 2). Then a CT scan demonstrated a round, well-marginated sclerotic lesion of about 8 mm of diameter with a hypodense rim and a centrally calcified nidus. This image was consistent with an osteoid osteoma (Figure 3).

### 2.1. Operative Technique

**Figure 4:**
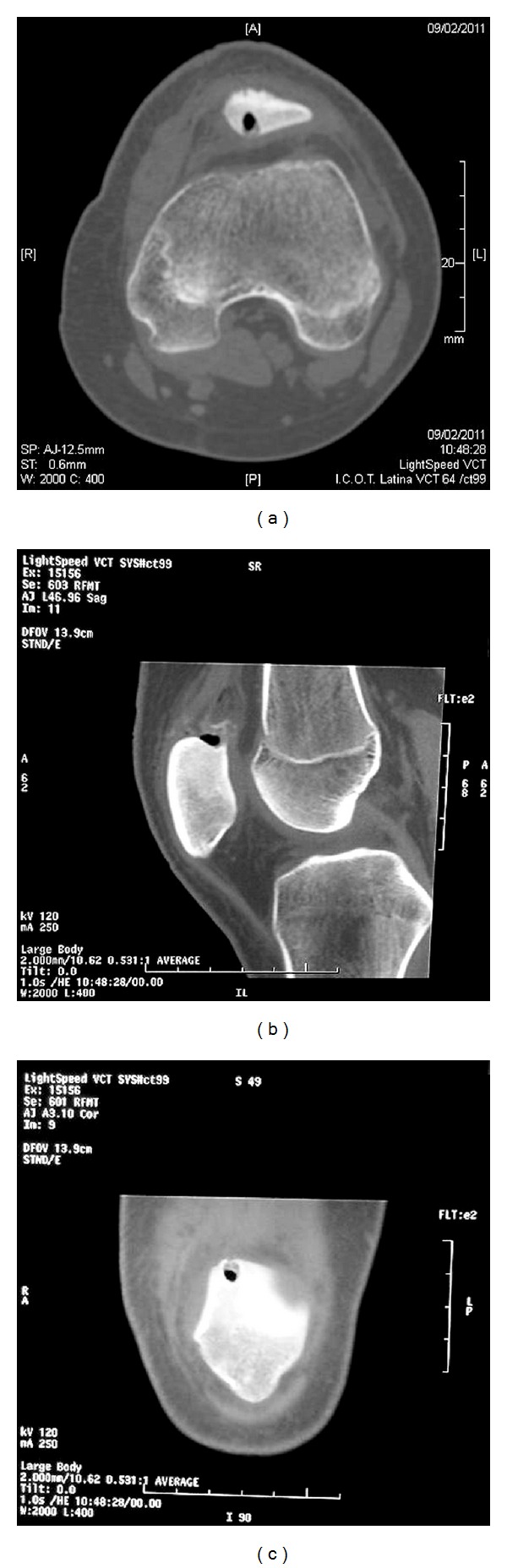
Postoperative CT scans confirm the disappearance of the nidus ((a) axial; (b) sagittal; (c) coronal view).

The chosen surgical treatment was a CT-guided percutaneous drilling. The procedure was performed in the CT suite under femoral nerve block and without the use of a tourniquet. A 3 mm Kirschner wire was initially driven percutaneously into the nidus under CT guidance. A superior approach through the quadriceps tendon was used in order to minimise retropatellar articular cartilage injury. Therefore, a small skin incision (1 cm) was made to accommodate a 8 mm drill that was inserted along the K-wire in order to remove the nidus mechanically. Postoperative CT scan confirmed that the nidus was entirely removed (Figure 4). 

### 2.2. Postoperative Care

 The patient was discharged the day after the operation. An antibiotic prophylaxis was given and the patient was suggested to use a knee sling for about two weeks. The pain disappeared immediately and completely since the night after surgery without any drugs consumption. The patient came back to the hospital for a checkup after four weeks, reporting no pain and a normal ROM of the knee joint. After 2 years of followup, the patient was still symptom free and no degenerative change of the patellofemoral joint was found.

## 3. Discussion

Osteoid osteoma is a benign and painful skeletal tumor that accounts for 10–12% of all benign bone tumors and 2,5% of all pediatric lesions [6, 7]. It occurs mainly in children and young adults with 90% of cases seen before the age of 25 years and a male/female ratio of more than 2 : 1 [8, 9]. It is typically a small lesion (less than 1,5 cm) composed by a central nidus of osteoid tissue surrounded by a rim of reactive sclerotic bone [8, 10]. Osteoid osteoma can occur everywhere in the skeleton both in the cortex and medulla [11]. However, it most commonly affects the lower extremity with 50% of all cases involving the femur or the tibia [12]. The characteristic clinical presentation is severe local pain which typically worsens at night and is usually relieved by aspirin and other nonsteroidal anti-inflammatory drugs [13]. Osteoid osteoma is usually diagnosable with plain radiographs which show the small radiolucent nidus surrounded by the typical reactive sclerotic rim. However, the periosteal reaction occurs in intracortical osteoid osteoma, but in intra articular or medullar osteoid osteoma sclerotic reaction may be totally absent [14]. Thus, the conventional radiological diagnosis may be difficult and a three-phase scintigraphy followed by CT or MR imaging may be necessary. Bone scintigraphy shows the typical double density sign, characteristic of osteoid osteoma [15], while MRI and CT usually provide help in confirming the diagnosis, characterizing the lesion and planning the treatment. Until recently, surgical excision was considered the standard treatment. However, since intraoperative localization of these small lesions can be very difficult, open surgical removal of the tumor often necessitates significant bone resection [16]. To overcome this problem several minimally invasive techniques have recently been developed. These include percutaneous excision with relatively large-caliber hollow needles and drills, MR-guided cryotherapy, arthroscopic removal, CT-guided drill resection, and percutaneous radiofrequency [17].

Patellar osteoid osteoma is rare and only few cases are described in the literature [13, 18–21]. It is a difficult lesion to diagnose, with misdiagnosis being very common and a resulting delay between the onset of symptoms and appropriate treatment [13]. Furthermore, unnecessary treatment is usually applied, such as use of crutches, knee braces, intra-articular injections, psychological evaluation, and finally arthroscopy. 

Koós and Than [22] reported a case of a 17-year-old girl with right anterior knee pain due to a patellar osteoid osteoma which was initially misdiagnosed with chondromalacia patella. After ineffective conservative therapy, arthroscopy was undertaken but was normal. Also Cohen et al. [19] described a case of a 25-year-old woman with a patellar osteoid osteoma who underwent 2 diagnostic arthroscopies and a femoral biopsy without any result. In our case the delay between the onset of symptoms and appropriate treatment was 6 months due to misdiagnosis as PFM. 

The conventional radiological diagnosis of patellar osteoid osteoma is difficult due to the absence of periosteal reaction [14]. Additionally, clinical features of osteoid osteoma may be present 6 to 8 months after onset of symptoms without any characteristic radiographic finding of the lesion [23]. Also in our case, the standard radiographs were negative but the typical clinical findings, and in particular the good response to nonsteroid antiinflammatory drugs regarding night pain, lead us to strongly suspect an osteoid osteoma that was later confirmed by bone scintigraphy and CT.

Different surgical procedures can be used for the treatment of a patellar osteoid osteoma. In addition to open and invasive techniques such as en bloc resection and Burr-Down technique, minimally invasive techniques as CT-guided percutaneous drilling are also used [8, 11, 24]. The surgical resection is difficult even under CT imaging because patella is a small bone, has lesser bone stock, and has a short distance between the anterior cortex and chondral surface [24]. In our case, an open surgical approach would have lead to the removal of the superior pole of the patella, where the lesion was located, resulting in a weakening of the quadriceps tendon insertion. Moreover, Vallianatos et al. [11] reported the development of patella infera due to the scarring and shortening of the patellar tendon as a consequence of the arthrotomy. Franceschi et al. [21] described a case of arthroscopic CT-guided resection of a patellar osteoid osteoma. However, the arthroscopic resection has the disadvantage of damaging the articular surface with the risk of developing patellofemoral osteoarthritis in the future.

Taking into account these considerations, we decided to perform a CT-guided percutaneous drilling that many studies have shown to be both safe and effective [9, 25]. This technique allowed us to have an accurate guidance, which is essential considering the mobility and small dimension of the patella, and to perform the resection with minimal bone loss and without damaging the articular cartilage.

In conclusion, patellar osteoid osteoma should be always included in the differential diagnosis for any adolescent presenting with anterior knee pain, especially when the pain is recurrent, nocturnal, and relieved by NSAIDS. 

Moreover, CT-guided percutaneous resection should be considered as the treatment of choice for this lesion although no histological examination is possible with this technique.
